# Thriving during COVID-19: Predictors of psychological well-being and ways of coping

**DOI:** 10.1371/journal.pone.0248591

**Published:** 2021-03-15

**Authors:** Ma. Teresa Tuason, C. Dominik Güss, Lauren Boyd

**Affiliations:** 1 Department of Public Health, University of North Florida, Jacksonville, Florida, United States of America; 2 Department of Psychology, University of North Florida, Jacksonville, Florida, United States of America; Middlesex University, UNITED KINGDOM

## Abstract

COVID-19 has led to global dramatic shifts in daily life. Following the biopsychosocial model of health, the goal of the current study was to predict people’s psychological well-being (PWB) during the initial lockdown phase of the pandemic and to investigate which coping strategies were most common among people with low and high PWB. Participants were 938 volunteers in the United States who responded to an online survey during the lockdown in April 2020. The main findings were that all three groups of variables, biological, psychological, and socio-economic, significantly contributed to PWB explaining 53% variance. Social loneliness and sense of agency were the strongest predictors. PWB was significantly predicted by physical health (not gender nor age); by spirituality, emotional loneliness, social loneliness, and sense of agency; by job security (not income, nor neighborhood safety, nor hours spent on social media). Comparing the coping strategies of participants, results show more intentional coping in the high-PWB group and more passive coping in the low-PWB group. During this unprecedented pandemic, the findings highlight that ability to sustainably cope with the global shifts in daily life depends on actively and intentionally attending to PWB by being one’s own agent for physical health, spiritual health, and social connection.

## Introduction

COVID-19 has changed the lives of people around the world in dramatic ways. The coronavirus was first detected in Wuhan, China in December 2019 and was initially named severe acute respiratory syndrome coronavirus 2 (SARS-CoV-2) [1]. On January 30, 2020, as the first cases were reported in Europe and in the United States, the World Health Organization named the Coronavirus disease 2019 (COVID-19) and declared it a public health emergency of global concern. Three months after COVID-19 was declared a global pandemic, in the United States there were 578,268 known COVID-19 cases and 23,476 deaths due to COVID-19 by April 15, 2020 [2]. Forty-two out of 50 states were on some sort of lockdown by the middle of April 2020 and most states lifted them in May. The current study was conducted in April 2020, right at the midst of the lockdown in the United States. Since then, the numbers have continued to rise; over 28 million people have contracted COVID-19 and over 500,000 people have died due to the virus in the United States as of February 22, 2021 [3]. State governments and health organizations continue to institute ways to respond to mitigate the spread of the virus, but equally important are people’s psychological well-being as they cooperate with these mitigation efforts. Moreover, while addressing the strictly biological component is critical, addressing the mental health of populations and understanding their coping behaviors through this period is equally crucial [4].

The goal of the current study is twofold: a) based on the biopsychosocial model of health [5], to investigate which variables best predict increased psychological well-being (PWB) during this pandemic, and b) to identify which ways of coping are associated with high PWB and low PWB.

Given its recency, only a few studies have researched the effects of COVID-19 on PWB. PWB refers to reaching one’s potential, being optimistic, having supportive social relationships, and a sense of purpose [6]. PWB was clearly affected in the phase of COVID-19 as studies in the United Kingdom and Spain have shown [7, 8]. Researchers have shown that PWB during the 2020 COVID-19 crisis is lower compared to PWB before COVID-19, with psychiatric symptoms such as anxiety, depressed mood, nervousness, and hopelessness increasing during COVID-19 [9]. In the United States, a poll found that 36% of Americans report that COVID-19 is seriously impacting their mental health, and 59% express significant impact of the virus in their everyday lives [10].

According to the biopsychosocial model of health, there might be multiple biological, psychological, and social factors that could affect PWB [5, 11]. We comprehensively investigated eleven predictors of PWB, that belong to three groups: biological, psychological, and social and economic variables. This is in response to one criticism of research on health and well-being where the focus is only on a few isolated variables at a time [12]. Health and well-being are multifaceted constructs with many causes. Considering the biological side alone will not adequately address the pandemic and the population’s well-being. Overcoming the pandemic places psychological burdens on the population and will also necessitate behavioral change [13]. Research on PWB during the COVID-19 outbreak requires concentrating on appraisals (psychological factors) and personal resources (social factors and economic factors) [14]. The biopsychosocial model is a framework that most aptly represents the multitude of variables influencing health. According to this model, only by considering biological, psychological, and social factors together will health and well-being be adequately explained [15–17]. Rather than looking at a few isolated variables, the model suggests looking at several variables together to assist in providing a more holistic understanding of subjective health phenomena. An approach based on this biopsychosocial model has also been advocated by many researchers focusing on COVID-19 treatment [18, 19].

### Biological factors (gender, age, physical health)

The biological variables we addressed in our study are gender, age, and physical health. Regarding gender, PWB tends to have more pronounced displays in women compared to men, with being female as a factor related to increased risk of mental health symptoms and lower PWB during COVID-19 [9] and even before COVID-19 [20]. Research showed that depressed mood, nervousness, and hopelessness were higher in women than men during the COVID-19 pandemic [21]. Even before COVID-19, studies showed that women had higher scores in expressing affect and men scoring higher on physical and total self-concept—findings which are in line with current socialization practices [22]. Other research shows the nuances of the impact of gender on PWB, being mediated by basic need satisfaction and job satisfaction [20].

The passage of time provides another biological component of PWB. Overall, PWB is positively associated with the progression of developmental goals with age [23]. However, there are notable factors which may lessen or change this relationship, such as culture [24] and socioeconomic status later in life [25]. Reasons for this increase in PWB with age have been tied to major psychological theories such as those proposed by Maslow [26] and Erikson [27]. Also during the initial months of the pandemic, older adults experienced less negative impact on their well-being [28, 29].

Outside biological factors such as gender and on-going changes related to age, PWB can also be a valuable determinant or buffer for physical health concerns. PWB has been found to serve as a protective factor both for disease and mortality risk [30], and for physical health ailments [31] such as cardiovascular disease [32]. Additionally, poor physical health has been found to be associated with lower PWB during COVID-19 [10].

### Psychological factors (spirituality, emotional loneliness, social loneliness, and sense of agency)

The psychological variables we included in our study are spirituality, emotional loneliness, social loneliness, and sense of agency. Spirituality can provide hope and meaning in difficult situations and adverse life events [33] including the coronavirus pandemic [34] and help with forgiveness of others and oneself [35, 36]. Spirituality as a regular routine and communal activity among older adults showed marked benefits towards PWB [37, 38]. The findings are, however, not consistent. Other studies, for example with Hurricane Katrina survivors, showed no effect of religiosity on stress symptoms [39].

Emotional loneliness, social loneliness, and sense of agency may be other important variables in the study of PWB during COVID-19 [13]. These variables are based on human needs described, for example, in Self-Determination Theory [40] and PSI-Theory [41]. Both theories refer first to the human need for affiliation and relatedness. People enjoy socializing and the physical presence of others. The current situation of COVID-19 with the directives of social distancing and isolation likely heightens the need for affiliation. People might experience loneliness as a consequence. Loneliness has been negatively related to PWB [42]. Emotional loneliness in our study refers to a feeling of emptiness and rejection.

Social loneliness refers to relationships with others, not having enough people to count on or to feel close to. Following the discrepancy-attributional approach, loneliness can be viewed as a “discrepancy between one’s desired and achieved levels of social relations” [43 p. 32, 44]. Especially during the pandemic, social loneliness and isolation are associated with an increase in experienced stress [13]. In relation to PWB, feelings of loneliness are detrimental throughout life and related to adverse psychological and physical health at each stage of life such as depression, poor sleep quality, compromised executive function, impaired immunity, and earlier mortality [45]. With the need to significantly decrease face-to-face contact, it comes as no surprise that early studies published during the COVID-19 pandemic found increased feelings of loneliness and social isolation [46]. Even in periods unrelated to disease, separation and lack of social contact predicted poorer PWB [47]. Loneliness is a critical factor in viewing detriments to PWB.

Another psychological variable of interest is the sense of agency, which is defined as the “I” being able to create, conduct, or cause an action [48]. Past research has shown a positive relationship between sense of agency and control with PWB [49]. The range of possible actions is restricted under stay-at-home orders and potentially the feeling of control and sense of agency might be low during COVID-19. High uncertainty also contributes to low feelings of control and sense of agency [50].

### Social factors (social media and neighborhood safety) and economic factors (job security and income)

We combined social and economic variables to study PWB. The two social variables studied were hours on social media and neighborhood safety. Access to social media offers an opportunity to interact with friends and family and could be a means to satisfy the need for affiliation. Thus, interacting on social media might decrease feelings of loneliness during the pandemic and the initial lockdowns. Earlier research done during the COVID-19 pandemic shows that social media usage increased in 40% of respondents [51]. Other research, however, found social media usage to be a risk factor for depression, poor sleep, neuroticism, and other detrimental effects [52]. While social media usage is not a homogenous construct and varies between programs and applications used, it is unclear what effects it has on PWB during COVID-19.

Perception of neighborhood safety was also evaluated. Although not directly related to neighborhood safety, research in Wuhan has shown that social infrastructure and neighborhood services decreased the negative impact of the COVID-19 outbreak on residents’ mental health [53]. Neighborhood safety was of particular concern to the PWB of elderly populations [54, 55]. While much research is focused on older-adult populations, there is evidence of neighborhood safety being a buffer to psychological distress across age groups [56].

Economic variables, such as job security and income, were added because during the pandemic many people lost their jobs and primary source of income. These personal resources have an impact on PWB during COVID-19 [14]. Additionally, previous research has shown the influence of economic factors on PWB, such as unemployment in the SARS epidemic [57], job stability [58], and income [59].

### Coping

Past research on coping with extreme events (e.g., getting paralyzed [60]; after a hurricane [61]) have shown that it is not so much the event per se that affects well-being, but more so the psychological processing of the event. Researchers have found that coping skills had a strong relationship to mental health [62] among health-care workers during this pandemic [63] and among the general population during this pandemic [64]. In a narrative synthesis of research that investigated coping responses in past outbreaks of infectious diseases, researchers identified four responses [65]: problem-focused coping (e.g., behaviors that empower them to stay healthy and have a sense of control), avoidance/denial/distraction (e.g., reading), positive appraisal (e.g., keeping a positive attitude), and seeking social support. Chew and colleagues remind the public that for this pandemic, coping strategies need to consider the individual in relation to their social context, for self-care and continued engagement [65]. As COVID-19 may be prolonged and protracted, sustainable coping strategies need to be identified among those whose PWB is optimal.

### Hypotheses

Based on the previous discussion we expect the following variables to be predictors of well-being during COVID-19: biological variables (being male, being older, and being physically healthy), psychological variables (being spiritual, not feeling lonely emotionally, not feeling lonely socially, and having a sense of agency), socioeconomic variables (less social media usage, living in a safe neighborhood, being employed, and having high income). We expect those with high PWB to use different coping strategies–especially those focusing on self-care, seeking social support, and creative potential to problem-solving—compared to those with low PWB.

## Method

### Participants

Participants were over 1,500 from 47 countries worldwide who voluntarily responded to the survey. We will focus our analyses solely on the 977 participants from the United States, because COVID-19 spread, developments, and circumstances differ widely between countries. Of the 977 U.S. participants’ data collected overall in the Qualtrics data file, 966 were complete; 11 participants completed all but three demographic questions at the end of the survey. All participants took longer than 240 seconds (i.e. 4 minutes) to take the survey which is a positive indicator for the quality of the data. We also checked for straightliners among the participants. Considering that the total response times were in a reasonable time frame and that almost all participants completed the whole survey, we decided to remove 15 participants who were straight-liners, i.e., had standard deviations of 0, on at least two of the surveys. We also removed 24 participants who were from the 5 states that had no restrictions at all (Arkansas—1, Iowa—2, Nebraska—6, North Dakota—1, South Dakota—0) and from the 3 states with regional/partial restrictions (Oklahoma—3, Utah– 9, and Wyoming—2). Thus, the final sample size was 938 participants, from 37 states and the District of Columbia. Participants were not compensated, although we promised to share the study’s results with those who were interested and provided their email addresses.

Regarding gender, out of 938 Americans, 648 (69.1%) were female, 267 (28.5%) were male, 6 (0.6%) were transgender, 12 (1.3%) were gender non-conforming, 1 (0.1%) identified as “Other,” 4 (0.4%) preferred not to answer. The participants’ age ranged from 18 to 82 (*M* = 37.34, *SD* = 13.41, N = 888).

Regarding ethnicity, 749 (79.9%) were White, 16 (1.7%) were Black or African American, 57 (6.1%) were Hispanic, Latino, or Spanish origin; 5 (0.5%) were Native American, American Indian, or Alaska Native; 57 (6.1%) were Asian or Asian American; 1 (0.1%) was Hawaiian or other Pacific Islander, 28 (3.0%) were Biracial or multiracial, 14 (1.5%) selected “Other,” and 11 (1.2%) were missing values.

### Instruments

Driving our decisions about the use of surveys to assess specific constructs were first, psychometric properties of the instruments (reliability and validity in previous studies), and second, brevity. We also followed common guidelines for creating internet surveys that avoid fatigue [66]. The complete survey and the data are available online under http://dx.doi.org/10.17504/protocols.io.brfcm3iw

#### Psychological well-being

PWB was assessed with an 8-item scale (e.g., “I lead a purposeful and meaningful life.” [67]). The advantage of this scale is that each of the eight items is related to a theoretical construct that has been linked to well-being in previous research: meaning and purpose, supportive and rewarding relationships, engaged and interested, contribute to the well-being of others, competency, self-acceptance, optimism, and being respected. Answer options were presented on a 7-point Likert scale ranging from “strongly agree” to “strongly disagree.” An overall mean score possibly ranging between 1.0 and 7.0 was calculated, with higher scores signifying higher PWB. Cronbach’s alpha reliability was .89. The reliability would not improve if any items were to be removed.

To calculate convergent validity, we also included one item assessing the subjective mental health overall (based on [68], AIOS): “Thinking of the last week, how is your overall well-being? Select the point on the line that summarizes your overall well-being for the last week.” A slider question was presented from 0 (“worst you have ever been”) to 10 (“best you have ever been”). The correlation between the overall mean score of the 8-item scale and the one-item question was *r* = .58 (*N* = 1240), *p* < .001 indicating convergent validity.

#### Biological variables

*Gender*. Gender was assessed with the question “What is your gender?” Answer options were female, male, transgender, gender non-conforming, other, prefer not to answer. For the hierarchical multiple regression analysis, we only included female (68.8%) and male (28.8%) as a dichotomous variable and coded the others together (2.4%) as missing values.

*Age*. Age was assessed with an open question “What’s your age?”

*Physical health*. We also assessed overall physical health with one question asking: “Thinking of the last week, how is your overall physical health? Mark the line below with an X at the point that summarizes your physical health for the last week.” A slider question was provided from 0—“worst you have ever been” to 10—“best you have ever been” [similar to the validated Arizona Integrative Outcomes Scale AIOS, 68]. Also the World Health Organization uses a similar one-item measure of health [WHOQOL-BREF, 69].

#### Psychological variables

*Spirituality*. We assessed spirituality with one question asking: “I consider myself to be a spiritual/religious person.” A slider question was provided from 0—“not at all” to 10—“very much” [similar to the one-item scale “How religious would you say you are?” 70]. Research on similar single-item religiosity scales has demonstrated their validity and reliability [71]. Other research states that “An ancillary but important note, i.e., in spite of the frequent critical comments on the single item self-rating scales to assess religiosity and SWB, they yield approximately the same associations as the multi-item comprehensive questionnaires” [72, p. 56].

*Emotional loneliness*. Emotional loneliness was assessed with the Loneliness Scale [73, 74] consisting of 3 items assessing for example “feeling of emptiness” and “feeling rejected” (overall score ranging from 0 to 3). None of these items explicitly included the word “loneliness.” Each item had three answer options, “no,” “more or less,” and “yes.” The survey has been applied successfully in seven other countries [74]. Following the coding mentioned by the survey’s authors, for the three negatively formulated items assessing emotional loneliness, an answer of “more or less” or “yes,” was counted as 1. Thus, the overall score for emotional loneliness could vary between 0 and 3. Cronbach’s alpha reliability was acceptable but not good with .588 for the emotional loneliness subscale (the reliability would not improve if we remove any of the three items).

*Social loneliness*. Social loneliness was assessed with the Loneliness Scale [73, 74] consisting of 3 items assessing for example “people that I can count on” or “enough people that I feel close” (overall score ranging from 0 to 3). None of these items explicitly included the word “loneliness”. Each item had three answer options, “no,” “more or less,” and “yes.” The survey has been applied successfully in seven other countries [74]. For the three positively formulated items assessing social loneliness, an answer of “no” or “more or less” was counted as 1. Thus, the overall score for social loneliness could vary between 0 and 3, with higher scores depicting higher levels of loneliness. Cronbach’s alpha reliability was .857 for the social loneliness subscale. The reliability would improve to .861 if we remove the third item; due to the low number of items we decided against removing it.

*Sense of agency*. Sense of agency was assessed with the Sense of Agency Scale [75] consisting of six items (e.g., “I can exercise my free will.”). Answer options were 9-point Likert scales from 1—“not at all” to 9—“a lot.” There were no reverse scored items. An overall mean of the six items was calculated for further analyses with higher scores denoting a higher sense of agency. Cronbach’s alpha reliability was .849 for the overall scale. The reliability would improve to .857 if we remove the first item, but due to the low number of items we decided against it.

#### Social and economic variables

*Hours communicating on social media*. We asked the question: “During an average day of the past week, how much time do you spend per average on social communication via smartphone, Facebook, and the many platforms?” A slider question was provided from 0 to 10 hours. Single items on social media use have been widely used such as “frequency of social media” from 1 “never” to 5 “very often” [76], but rather than a subjective measure we preferred an objective single item on behavior measuring time online such as the one used in other research [77, 78].

*Neighborhood safety*. Neighborhood safety was assessed with the question: “How safe would you say is your neighborhood?” A slider question was provided from 0—“not safe at all” to 10—“very safe.” A similar one-item safety scale was used in other research on neighborhood safety [79, 80]. Single items on neighborhood safety have also been validated [81].

*Job security*. We included one question assessing current job security. “Job: I am currently employed (select 1 or 2): In paid employment; Self-employed; Part-time work; Unemployed, looking for work; Uncertain, e.g. awaiting company decision; Looking after family/home; Student; Retiree; Other;” We recoded the answers to this question as 1 –unemployed or looking for work, 2 –part-time work or uncertain (awaiting company decision, student, or uncertain self-employed), and 3 –in paid employment or retiree. Looking after family and other was coded as missing value.

*Income*. Income was assessed with the question: “Thinking of your household’s total monthly income NOW, is your household able to make ends meet *now*?” [82, 83]. A slider question was provided from 0—“with great difficulty” to 10—“very easily.”

#### Coping behavior related to COVID-19

We created a new coping scale that is tailored to the COVID-19 pandemic, but is also informed by the perspective of social connection, interpersonal relationships, and healthy attitudes as proposed by scientists discussing the social and behavioral research for the response to COVID-19 [13]. Our focus in this study was on concrete behaviors in daily life rather than subjective and more abstract rating questions. A question was created by the authors asking “What do you like/enjoy in your current situation? Read through the list first and then select 5 things you enjoy among the following.” This question was initially presented as an open-ended question to a sample of 10 student volunteers. Based on their responses and the thoughts of the authors, all items were combined into the 23 answer options for coping strategies (see Table 3 for the 23 items).

### Procedure

This study was approved by the University of North Florida Institutional Review Board (#1589677,”Coronavirus Survey 1”). Every participant either agreed to participate or not after reading the written informed consent. We planned a “rapid” study to gather data during the lockdown of COVID-19, when the most stringent measures of physical distancing, stay-at-home orders, and limiting work to essential services were still in place in 42 states of the United States during April 2020. We started data collection April 15, 2020 and we ended data collection on May 2, 2020 for this study.

The 40-question survey was briefly introduced on various social media websites as a “study to learn from respondents about how COVID-19 is affecting their overall life and well-being.” The survey link was posted on internet sites such as Reddit (on 28 different subreddits all related to either the coronavirus, psychological studies, or scientific research), Tumblr, and on Facebook. After giving their consent, participants responded to the survey which took around 12 minutes to complete.

### Data analysis

Data were collected via a Qualtrics-link and downloaded in Excel. Further analyses were conducted using the IBM SPSS Statistics 27 software. First, we conducted Pearson correlation coefficients among all variables and then a hierarchical regression with PWB as outcome. Step 1 included the biological variables, step 2 the psychological variables, and step 3 the social and economic variables. Additionally, we conducted regression analyses for the three groups of variables separately. For all analyses, bootstrapping with 1000 samples was used to account for non-normality or multi-collinearity of variables and outliers. To analyze the coping strategies during COVID-19, we conducted extreme group comparison based on PWB scores. The lowest and highest tertiary PWB group were compared.

To determine minimum sample size, we run a power analysis in G*Power [84] for a medium effect size of f^2^ = 0.15 [85], an alpha level of 0.01, with a power of 0.95, and a total number of 11 predictors. The program showed a minimum sample size of 227, which was easily achieved.

## Results

### Hierarchical multiple regression predicting PWB

Bootstrapped Pearson correlations among variables are shown in Table 1. For the hierarchical regression first, we entered the three biological variables: gender, age, and physical health to predict PWB. Second, we added the four psychological variables: spirituality, emotional loneliness, social loneliness, and sense of agency. Third, we entered the four social and economic variables: hours communicating on social media, neighborhood safety, job security, and income.

**Table 1:** Descriptive statistics and bootstrapped pearson correlations for all variables.

	*M*	*SD*	Psych well-being	Gender	Age	Physical health	Spirituality	Emotional loneliness	Social loneliness	Sense of agency	Hrs. communicating	Neighborhood safety	Job security
PWB	5.05	1.14											
Gender			-.07*										
Age	37.34	13.41	.23***	-.13***									
Physical health	5.81	2.21	.40***	.02	.12**								
Spirituality	4.28	3.57	.31***	-.13***	.32***	.17***							
Emotional loneliness	1.70	0.93	-.33***	-.007	-.22***	-.17***	-.02						
Social loneliness	1.40	1.28	-.52***	.08*	-.06	-.18***	-.19***	.17***					
Sense of agency	7.06	1.39	.54***	-.009	.27***	.30***	.16***	-.33***	-.25***				
Hours communica-ting on social media	4.36	2.75	-.05	-.16***	-.06*	-.09**	.14***	.13***	-.01	-.02			
Neighbor-hood safety	8.13	2.06	.26***	-.06	.14***	.20***	.17***	-.13***	-.23***	.20***	.02		
Job security	2.53	0.62	.21***	-.03	.22***	.02	.05	-.17***	-.13***	.13***	-.06	.04	
Income	7.50	2.67	.26***	.02	.14***	.23***	.10*	-.18***	-.21***	.16***	-.11*	.26***	.35***

*Note*. PWB = Psychological Well-Being, Gender 1 = female, 2 = male.

* *p* < .05.

** *p* < .01.

*** *p* < .001.

The three biological variables alone explain 19.7% of the variance in PWB, *F*(3, 863) = 70.76, *p* < .001. Age and physical health were significant predictors; gender was not. Adding the four psychological variables significantly improved the model and explained 52.2% of the variance in PWB (an additional 32.2%), *F*_change_(4, 848) = 143.77, *p* < .001. The effect size attributable to the addition of the psychological variables to the model, Cohen’s f^2^ = 0.680, indicates a large effect. The four psychological variables spirituality, emotional loneliness, social loneliness, and sense of agency were all significant predictors. The overall model with the 7 variables was significant, *F*(7, 848) = 132.12, *p* < .001. When conducting regression analyses separately and only using the four psychological variables as predictors, they were also all significant predictors of PWB (*p*s < .001), R^2^ = .499, *F*(4, 922) = 229.86, *p* < .001.

Adding the four social and economic variables in the third step further improved the model significantly and explained 53.3% of the variance in PWB (an additional 1.2%), *F*_change_(4, 768) = 4.89, *p* = .001. The effect size attributable to the addition of the social and economic variables to the model, Cohen’s f^2^ = 0.026, indicates a small effect. Only job security was a significant predictor. The overall model with the 11 variables was significant, *F*(11, 768) = 79.74, *p* < .001. When conducting regression analyses separately and only using the four social and economic variables as predictors, neighborhood safety, job security, and income were significant predictors of PWB (*p*s < .001), R^2^ = .131, *F*(4, 839) = 31.73, *p* < .001.

Looking at the final model (see Table 2), among the biological variables, gender and age were not significant predictors, only physical health was. All four psychological variables spirituality, emotional loneliness, social loneliness, and sense of agency were significant predictors. Among the four social and economic variables, income, neighborhood safety, and hours on social media were not significant predictors, but job security was a significant predictor. Sense of agency and social loneliness were the strongest predictors.

**Table 2:** Hierarchical regression results for psychological well-being, bootstrapped.

Variable	B	95% CI	for B	SE B	β	*R*^2^	Δ*R*^2^
		*LL*	*UL*				
Step 1: Biological variables						.20***	
Constant	3.50	3.17	3.84	.18			
Gender	-.11	-.26	.04	.08	-.04		
Age	.02	.01	.02	.003	.18***		
Physical health	.20	.17	.23	.02	.38***		
Step 2: Biological and psychological variables						.52***	.32***
Constant	2.93	2.49	3.40	.23			
Gender	-.10	-.17	.08	.06	-.02		
Age	.002	-.003	.01	.002	-.02		
Physical health	.10	.07	.13	.01	.19***		
Spirituality	.04	.03	.06	.01	.14***		
Emotional loneliness	-.13	-.19	-.08	.03	-.11***		
Social loneliness	-.31	-.35	-.25	.02	-.35***		
Sense of agency	.29	.24	.33	.03	.34***		
Step 3: + socio-economic variables						.53***	.01***
Constant	2.45	1.88	2.98	.27			
Gender	-.06	-.19	.05	.06	-.02		
Age	.001	-.005	.005	.002	-.001		
Physical health	.09	.06	.12	.01	.18***		
Spirituality	.05	.03	.06	.01	.14***		
Emotional loneliness	-.12	-.19	-.05	.03	-.10***		
Social loneliness	-.29	-.33	-.23	.02	-.32***		
Sense of agency	.28	.23	.33	.03	.34***		
Hours communicating on social media	-.02	-.04	.01	.01	-.04		
Neighborhood safety	.02	-.01	.05	.02	.04		
Job security	.15	.05	.26	.05	.09**		
Income	.01	-.01	.04	.01	.03		

*Note*. CI = BC Confidence interval, LL = lower limit, UL = upper limit. Gender was coded as 1 = female and 2 = male.

* *p* < .05,

** *p* < .01,

*** *p* ≤ .001

### Qualitative data on coping during the 2020 COVID-19 pandemic

Table 3 shows the 23 coping items: what participants like/enjoy in their current situation. We will discuss only the most frequent coping items (endorsed with a percentage of over 30%) for the low-PWB group and the high-PWB group, and discuss the coping strategies where the two groups significantly differ. In order to proceed with the analysis, we classified the participants into three groups according to the overall mean in PWB, lowest with scores from 1.0 to 4.63 (n = 295), medium with scores from 4.64–5.63 (n = 322), and highest with scores from 5.64 to 7.0 (n = 321). The comparisons refer only to the extreme groups between the lowest 1/3 and the highest 1/3 of all participants, according to their PWB levels.

**Table 3:** Frequencies and chi-square results for coping behaviors in low- versus high-PWB groups.

Variable	Low PWB (*n* = 295)		High PWB (*n* = 321)		*χ*2 (1)
	n	*%*	*n*	*%*	
Having more time with family or people I live with	81	27.5	186	57.9	58.18***
Having more time on social media/Non-face-to-face communication	28	9.5	14	4.4	6.37*
My current housing situation	52	17.6	73	22.7	2.49
Having more time for myself to rest/reflect/re-energize/slow down	100	33.9	146	45.5	8.60**
Not having to drive/go/commute around so much	121	41.0	101	31.5	6.09*
The outdoors, nature, the environment	66	22.4	103	32.1	7.29**
More time with daily living: cooking, cleaning, organizing	80	27.1	97	30.2	0.72
Getting to spend more time playing video games	65	22.0	27	7.8	25.01***
Getting to spend more time watching TV shows/movies	64	21.7	33	10.3	15.10***
Getting to spend more time relaxing with my pet	87	29.5	68	21.2	5.63*
Getting projects done around the house	49	16.6	115	35.8	29.06***
I’m not spending a lot of money	141	47.8	146	45.5	0.33
Having fewer responsibilities	76	25.8	43	13.4	15.09***
Having efficient/working technology to continue work/school	47	15.9	58	18.1	0.50
Having a richer spiritual/inner life	21	7.1	39	12.1	4.43*
Ability to work from home–I know a lot of people can’t	79	26.8	126	39.3	10.77***
Having more time to catch up on work /schoolwork	23	7.8	21	6.5	0.37
Since I work in an essential workplace, more time to work and make money	22	7.5	28	8.7	0.33
Having more time for hobbies/ entertainment (e.g., painting, boardgames, baking)	83	28.1	67	20.9	4.40*
Being creative and finding new ways to have fun	37	12.5	43	13.4	0.10
Having more time for exercise and physical activity	38	12.9	52	16.2	1.36
Nothing	64	21.7	8	2.5	54.92***
Other	46	15.6	11	3.4	27.10***

* *p* < .05

** *p* < .01.

*** *p* < .001.

We report coping mechanisms that both groups endorsed at more than 30%. Individuals in the low-PWB group, identified their most frequent coping strategies as “I’m not spending a lot of money” (47.8%), “Not having to drive/go/commute around so much” (41.0%), and “Having more time for myself to rest/reflect/re-energize/slow down” (33.9%). On the other hand, those in the high-PWB group named their most frequent coping mechanism as: “Having more time with family or people I live with” (57.9%), “I’m not spending a lot of money” (45.5%), “Having more time for myself to rest/reflect/re-energize/slow down” (45.5%), “Having the ability to work from home–I know a lot of people can’t” (39.3%), “Getting projects done around the house” (35.8%), and “Not having to drive/go/commute around so much” (31.5%), “The outdoors, nature, the environment” (32.1%), and “More time with daily living: cooking, cleaning, organizing” (30.2%).

Using the frequencies of the endorsed coping strategies by each group, we used the Chi-square to test whether these differences are significant, as seen in Table 3. The following differences between the low and high-PWB group are significant with *p* < .01. The low-PWB group, much more than the high-PWB group, significantly endorsed four coping strategies: “Getting to spend more time playing video games,” “Getting to spend more time watching TV shows/movies,” “Having fewer responsibilities,” and “Nothing.”

The high-PWB group, much more than the low-PWB group, significantly enjoyed these five ways of coping: “Having more time with family or people I live with,” “Having more time for myself to rest/reflect/re-energize/slow down,” “Getting projects done around the house,” “The outdoors, nature, the environment,” and “Having the ability to work from home–I know a lot of people can’t.”

## Discussion

The overall goal of the current study was to investigate PWB during the COVID-19 pandemic two months after the disease outbreak was declared a global public health emergency by the WHO on January 30, 2010 (WHO, 2020). Following the biopsychosocial theoretical model [5, 11], a specific objective was to comprehensively analyze eleven predictors altogether that belong to three groups: biological, psychological, and social and economic variables; and a second objective was to study coping and life changes.

The first group of biological variables explained 19.7% of variance in PWB. Including the psychological variables added significantly to the model and explained an additional 32.2% of the variance in PWB. Finally, including the socioeconomic variables explained an additional 1.2% of the variance in PWB. The findings show that all groups of variables are important and meaningful when predicting PWB and add to the validity of the biopsychosocial theoretical model [15–17]. Whereas past research on well-being has often focused only on very few variables at a time, the strength of the current study is investigating the complex set of variables. This examines the relative contribution of each variable when predicting PWB during COVID-19, in conjunction with the other predictors.

The overall model, which explains 53.3% of the variance, identifies the six significant predictors for PWB during the 2020 pandemic of COVID-19, in order of significance from most to least as: sense of agency, social loneliness, physical health, spirituality, emotional loneliness, and job security. Several of these PWB predictors, taken separately, have also been demonstrated in previous research before COVID-19. Sense of agency is related to the need for competence, experiencing that one’s actions can have the intended effects [41]. People initially experienced being at the mercy of COVID-19, something they can hardly control [86]. Social loneliness highlights the need for affiliation and relatedness [40, 41] as highly active. Social connectedness helps people cope with the stress related to the pandemic [13]. Physical health, specifically, exercise and overall engaging in physical activity, was found to be significantly associated with PWB or other mental health outcomes [87]. In terms of the impact of spirituality, our findings support another study that found that when it is more utilized in its positive form (e.g., religious coping, religious identity commitment), spirituality is associated with greater health and coping [34, 88]. Job security is identified as a predictor of mental well-being [89], and its negative relationship with mental health is even more pronounced during economically turbulent times [90] and during this pandemic [91]. During COVID-19, with many businesses closing and the uncertainty of the markets and the future overall, our study found that being able to rely on a stable job was an important contributing factor for PWB.

In terms of coping and changes in life, the low-PWB group significantly reported more passive coping strategies: time playing video games, watching TV, having fewer responsibilities, and “doing nothing.” However, playing video games and watching TV, are coping strategies that are not as beneficial, because frequent use of digital media was associated with lower PWB [92]. Moreover, an increased use of the internet was associated with lessened communication with family members in the household, a smaller social circle, and increased loneliness and depression [93]. Gaming or watching TV are ways of coping that may provide distraction or avoidance [65]. However, they may, in essence, decrease satisfaction with life and with individuals’ relationships, and increase symptoms of depression [94].

On the other hand, the high-PWB group utilized more intentional coping strategies that provide an opportunity for exercising a sense of agency, like getting projects done around the house and enjoying the ability to work from home. Intentional coping can be defined as mindful and proactive coping. The high-PWB group more frequently enjoyed having more time with family or people they live with; having time for themselves whether that is to rest, reflect, re-energize, or slow down; and the outdoors and nature. All of these reflect coping strategies in previous research on infectious diseases, especially positive appraisal and seeking social support [65]. Other research has shown that having a few strong relationships with others is key to health and well-being [95]. The important role of social support was also demonstrated in a study investigating well-being in a sample highly affected by the Chernobyl disaster [96]. In our study, potentially enjoying relationships and being more intentional about how time is spent may make participants feel less lonely, have a greater sense of agency and control, and serve as a buffer to satisfy the need for certainty. Also, other research on coping during the COVID-19 pandemic has shown that, for example, autonomy and purpose in life predict PWB [97] and pursuing hobbies and staying outdoors were predictors of fewer depressive symptoms [64].

## Implications

Especially because pharmaceutical interventions are not coming as fast as we need them, what we learn from research in the social and behavioral sciences can inform the way we manage the effects of this pandemic [13]. It is very likely that this period of surviving and responding will take time, and it behooves us all to respond in ways that are sustainable. Governmental restrictions and lockdowns are meant to be protective of the self and protective of the most vulnerable [98]. However, lockdowns can still take a toll on people’s PWB, where they can strongly impact the social need of people to be with friends and/or family. Which factors can sustain us and offer support for our psychological well-being?

Our findings show that the strongest predictors of PWB, the ones that matter the most in sustaining people’s PWB even with restrictions and lockdowns, are a sense of agency during these uncertain times, alongside with experiencing low social loneliness during COVID-19. The key to making this period sustainable may be owning the power, to whatever extent possible, in terms of being one’s own agent and being able to make one’s own choices, especially pertaining to being connected socially with others. Such perceived power in one’s agency was also found in individuals who adopted strategies to physically distance [99]. These predictors to PWB may just be the psychological support strategies that can help individuals cope with losing some freedoms for the greater good of all [100].

With the significant differences between low and high PWB coping, our study clearly delineates ways of coping under this global pandemic condition. These specific ways of coping are what may mitigate consequences to people’s mental health under COVID-19 [4]. We exercise self-agency when we undertake projects in our homes such as cleaning closets, painting, organizing photos; or when we take time for ourselves to rest, re-energize and reflect such as journaling, taking naps, eating mindfully; or when we enjoy more time with family such as playing together, cooking together, reading together, or phoning or mending relationships; or when we spend time outdoors such as taking a walk, watching the sun set, listening to the birds. Another concrete implication is that despite physically distancing, we could still decrease social loneliness, when we are intentional with the use of our time in our relationships with self (reflecting, resting) and others (telephone calls, writing texts or letters). Such greater sense of agency and control gives us a psychological boost until the time when the pharmaceutical boosts are adequate and available.

Limitations of the current study are related to sample and online survey methodology. First, although the sample size is quite large, it is still a cross-sectional convenience sample and not a representative sample, and not a sample stratified by age, gender, or race. Second, we discussed gender differences under biological variables. Strictly speaking, we did not discuss biological sex differences. Even though the question on gender might be understood by most participants as related to sex, gender can be also understood as a construct influenced by the socio-cultural environment. Third, we used an online survey and therefore had to restrict its length and use several single items to assess constructs. We did this intentionally, however, to avoid possible survey fatigue with long online surveys. One problem, for example, was related to the 3-item emotional loneliness scale which had a relatively low reliability of .588. Future research could use longitudinal designs to assess PWB over the course of this pandemic.

## Conclusions

COVID-19 is a health crisis that requires dramatic behavioral changes in the population. Thus, it is necessary to look at findings from the behavioral and social sciences [13]. To sum up the findings of the current study, the coping strategies that characterize individuals who are thriving despite COVID-19 are congruent with the identified predictors of PWB. Optimal PWB coping strategies include intention and purposiveness, finding new things to enjoy in caring for one’s physical and spiritual health and cultivating relationships with family, friends, and with oneself. Such ways of coping substantiate how sense of agency and social loneliness are the strongest predictors of PWB. Our results show the resourcefulness of people to not just cope with the challenges and the uncertainty of the COVID-19 pandemic, but to move forward and thrive.

## Supporting information

S1 File (DOCX)

S1 Data (SAV)
