# Supplementary material for: Thriving during COVID-19: Predictors of psychological well-being and ways of coping
Source: PLoS One. 2021 Mar 15;16(3):e0248591. doi: 10.1371/journal.pone.0248591 (PMC7959390; doi:10.1371/journal.pone.0248591)
Supplement: S1 File — (DOCX) [file pone.0248591.s001.docx]

Coronavirus Survey

Start of Block: Block 1

Q2
Informed Consent for “Living with the Coronavirus”
  January 30, 2020, the World Health Organization declared the COVID-19 crisis a global health emergency. February 28, 2020, the first coronavirus patient dies in the United States. Now it is April, and worldwide, thousands have lost their lives due to the coronavirus.   The University of North Florida invites you to participate in this study on COVID-19, if you are 18 years of age or older. The main purpose of this research study is to gain a deeper understanding of how individuals experience the current crisis. Should you choose to participate, you will be asked to answer a total of 40 questions related to your experience. The survey will take approximately 12 minutes. You may benefit from participation in the study by reflecting more deeply about the current situation and what it means to you.   It is important for you to understand that you will not be judged in any way during the study.  All your reports will be kept anonymous and secure in password protected files. Only the principal researcher, Dr. Dominik Guess and his research assistants will have access to the anonymous survey responses. A non-identifiable number will be used to mark your particular survey data, and this number will not connect to you in any way, unless you are willing to provide your email address at the end of the survey and indicating it is ok to contact you for possible follow-up surveys.   The experiences in this study are relatable to experiences in daily life and we do not expect foreseeable risks from this study. Due to the topic, however, there is a possibility that some participants may experience anxiety or emotional distress as a result of completing the survey. If you may be negatively impacted by participating in the survey, feel free to contact the CDC website for help under http://www.cdc.gov/coronavirus/2019-ncov/daily-life-coping/managing-stress-anxiety.html   If you have any questions about this research, feel free to contact Dr. Christoph Dominik Guess by phone: (904) 620-1634 or e-mail: dguess@unf.edu. If you have questions about your rights as a volunteer in this research project, please contact the UNF Institutional Review Board, (904) 620-2498 or send an e-mail to irb@unf.edu.   Participation in this study is completely voluntary. Refusal to participate or withdrawal will involve no penalty. If you feel uncomfortable, you may discontinue participation at any time, for any reason. 
 
Thank you for your participation in this study. Please print a copy of this form for your records.  
  
Sincerely,
Lauren Boyd &
C. Dominik Guess
Ma. Teresa Tuason
Professors, University of North Florida


 
By clicking on yes, you attest that you are at least 18 years of age and agree to take part in this research study.
 
By clicking on no you will not participate.

- Yes (1)
- No (2)

End of Block: Block 1

Start of Block: Default Question Block

Currently, most states are in stay-at-home orders to prevent the further spread of the COVID-19 virus. Most all of us stay home, keep social distance, and avoid crowds.

| 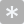 |
| --- |

Q1 What are currently your biggest concerns?

Please read through the list first and then select your 5 main concerns among the following:

- My own health (1)
- The health of anyone I love (2)
- Accidentally infecting others (3)
- My current housing/ living situation (e.g., not affording it, not getting along) (4)
- Not having enough supplies (e.g., food, medical, hygiene) for my everyday (5)
- Not having enough supplies in my community (6)
- Other countries around the world not having enough supplies (7)
- Other people not doing social distancing (8)
- Healthcare professionals and medical communities in extreme stress (9)
- The economy crashing (10)
- Possible violence or chaos (11)
- Not having income (12)
- Having to continue to work because my job can not be done remotely (13)
- Working/ going to school remotely (14)
- The uncertainty of the entire situation (15)
- My children's school, future, health (16)
- Other _______ (see next question) (17)

Q2 If you selected other, please type here what other big concern(s) you have:

________________________________________________________________

________________________________________________________________

________________________________________________________________

________________________________________________________________

________________________________________________________________

| Page Break |  |
| --- | --- |

| 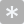 |
| --- |

Q3 Thinking of your *daily routine* now, what do you currently miss compared to your life several weeks before the coronavirus crisis?

Please read through the list first and then select 5 things you miss among the following:

- My freedom to go and do whatever I want (1)
- Face to face interactions with people (2)
- Certainty, health, and security: not being stressed about getting sick (3)
- Housing conditions, where I lived before the crisis started (5)
- My usual routine: going to work/ school (6)
- I miss giving hugs/receiving them (7)
- Going for leisure activities: restaurants, bars, shopping (8)
- Going to the gym, beach (9)
- Other _____ (4)

Q4 If you selected other, please type here what else you miss compared to before the coronavirus crisis:

________________________________________________________________

________________________________________________________________

________________________________________________________________

________________________________________________________________

________________________________________________________________

| Page Break |  |
| --- | --- |

| 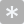 |
| --- |

Q5 What do you like/enjoy in your current situation?

Please read through the list first and then select 5 things you enjoy among the following:

- Having more time with family or people I currently live with (1)
- Having more time on social media/ Non-face-to-face-communication (2)
- My current housing situation (3)
- Having more time for myself to rest/ reflect/ re-energize/ slow down (5)
- Not having to drive/ commute so much (6)
- The outdoors, nature, the environment (7)
- More time with daily living: cooking, cleaning, organizing (8)
- Getting to spend more time playing video games (9)
- Getting to spend more time watching TV shows/movies (10)
- Getting to spend more time relaxing with my pet (11)
- Getting projects done around the house (12)
- I’m not spending a lot of money (13)
- Having fewer responsibilities (14)
- Having efficient working technology to continue work/ be in school (15)
- Ability to work from home – I know a lot of people can’t (17)
- Having more time to catch up on work / schoolwork (18)
- Since I work in an essential workplace, more time to work and make money (19)
- Having more time for hobbies/ entertainment (e.g., painting, board games, baking) (20)
- Being creative and finding new ways to have fun (21)
- Having more time for exercise and physical activity (22)
- Having a richer spiritual/ inner life (16)
- Nothing (23)
- Other ___________ (24)

Q6 If you selected other, please type here what else you like/enjoy in your current situation:

________________________________________________________________

________________________________________________________________

________________________________________________________________

________________________________________________________________

________________________________________________________________

| Page Break |  |
| --- | --- |

Q7 *Thinking of the last week,...*

|  | No (1) | More or less (2) | Yes (3) |
| --- | --- | --- | --- |
| 1. I experience a general sense of emptiness more-so than before the quarantine. (1) |  |  |  |
| 2. I miss having people around. (2) |  |  |  |
| 3. Often, I feel rejected. (3) |  |  |  |
| 4. There are plenty of people that I can lean on in case of trouble. (4) |  |  |  |
| 5. There are many people that I can count on completely. (5) |  |  |  |
| 6. There are enough people that I feel close to. (6) |  |  |  |

| Page Break |  |
| --- | --- |

Q13 Overall physical health
 Thinking of the last week, how is your overall physical health?
Select the point on the line that summarizes your **overall sense of physical health** for the last week.

|  | Worst you have ever been | Best you have ever been |
| --- | --- | --- |

|  | 0 | 10 |
| --- | --- | --- |

| Physical health () | 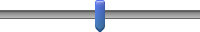 |
| --- | --- |

Q14 **Overall well-being** Thinking of the last week, how is your overall well-being?
Select the point on the line that summarizes your **overall sense of well-being** for the last week.

|  | Worst you have ever been | Best you have ever been |
| --- | --- | --- |

|  | 0 | 10 |
| --- | --- | --- |

| Well-being () | 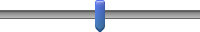 |
| --- | --- |

| Page Break |  |
| --- | --- |

Q15
**Your Opinions**
 
  Below are 8 statements with which you may agree or disagree. Considering the **last week** and using the 1–7 scale below, indicate your agreement with each item by indicating that response for each statement.

|  | Strongly disagree (18) | Disgree (19) | Somewhat disagree (20) | Neither agree nor disagree (21) | Somewhat agree (22) | Agree (23) | Strongly agree (24) |
| --- | --- | --- | --- | --- | --- | --- | --- |
| 1. I lead a purposeful and meaningful life. (1) |  |  |  |  |  |  |  |
| 2. My social relationships are supportive and rewarding. (2) |  |  |  |  |  |  |  |
| 3. I am engaged and interested in my current daily activities during quarantine. (3) |  |  |  |  |  |  |  |
| 4. I actively contribute to the happiness and well-being of others. (4) |  |  |  |  |  |  |  |
| 5. I am competent and capable in the activities that are important to me. (5) |  |  |  |  |  |  |  |
| 6. I am a good person and live a good life. (6) |  |  |  |  |  |  |  |
| 7. I am optimistic about my future. (7) |  |  |  |  |  |  |  |
| 8. People respect me. (8) |  |  |  |  |  |  |  |

| Page Break |  |
| --- | --- |

Q23
Below are 6 statements with which you may agree or disagree. Considering the **last week** and using the 1–9 scale below, indicate your agreement with each item by indicating that response for each statement.

|  | 1 - Not at all (97) | 2 (98) | 3 (99) | 4 (100) | 5 (101) | 6 (102) | 7 (103) | 8 (104) | 9 - A lot (105) |
| --- | --- | --- | --- | --- | --- | --- | --- | --- | --- |
| 1. I have a distinct voice/perspective. (1) |  |  |  |  |  |  |  |  |  |
| 2. I can exercise my free will. (2) |  |  |  |  |  |  |  |  |  |
| 3. I have control over my own voice. (3) |  |  |  |  |  |  |  |  |  |
| 4. I have the ability to assert myself. (4) |  |  |  |  |  |  |  |  |  |
| 5. I have control over my actions. (5) |  |  |  |  |  |  |  |  |  |
| 6. I have control over the information I consume. (6) |  |  |  |  |  |  |  |  |  |

| Page Break |  |
| --- | --- |

| 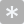 |
| --- |

Q29
Demographics Questions


Age

________________________________________________________________

Q30 Gender

- Female (1)
- Male (2)
- Transgender (3)
- Gender non-conforming (4)
- Other (5)
- Prefer not to answer (6)

Q31 What’s your sexual orientation?

- Heterosexual or straight (1)
- Gay (2)
- Lesbian (3)
- Bisexual (4)
- Other (5)
- Prefer not to answer (6)

Q32 In which country do you currently live?

________________________________________________________________

Q33 Which of the following best describes your current living situation?

- Live alone in my own home (1)
- Live alone in my own home with a pet (2)
- Live in a household with family - both parents and kid(s) (3)
- Live in a household with family - single-parent with kid(s) (4)
- Temporarily staying with a relative or friend (5)
- Live in a nursing home or any kind of hospital (6)
- Temporarily staying in a shelter or homeless (7)
- Other (8)
- Live with partner or roommate (9)

| 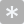 |
| --- |

Q34 What are the ages of the people living in the household together right now?

________________________________________________________________

________________________________________________________________

________________________________________________________________

________________________________________________________________

________________________________________________________________

Q35 Job situation

- In paid employment (1)
- Self-employed (2)
- Part-time work (3)
- Unemployed, looking for work (4)
- Uncertain, e.g. awaiting company decision (5)
- Looking after family/home (6)
- Student (7)
- Retiree (8)
- Other (9)

| Page Break |  |
| --- | --- |

Q36 Ethnicity

- White (1)
- Black or African American (2)
- American Indian or Alaska Native (3)
- Hispanic, Latino or Spanish origin (4)
- Native Hawaiian or Pacific Islander (5)
- Asian or Asian American (6)
- Biracial or multiracial (7)
- Other (8)

Q37 I consider myself to be a spiritual/religious person:

|  | Not at all | Very much |
| --- | --- | --- |

|  | 0 | 10 |
| --- | --- | --- |

| Spiritual () | 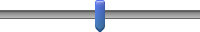 |
| --- | --- |

Q38 Thinking of your household’s total monthly income NOW is your household able to make ends meet *now*?

|  | With great difficulty | Very easily |
| --- | --- | --- |

|  | 0 | 10 |
| --- | --- | --- |

| Make ends meet () | 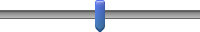 |
| --- | --- |

Q39 How safe would you say is your neighborhood?

|  | Not safe at all | Very safe |
| --- | --- | --- |

|  | 0 | 10 |
| --- | --- | --- |

| Safety () | 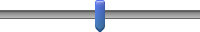 |
| --- | --- |

Q40 How easy is it for you to get the following resources you may need usually? (If there are other resources not yet mentioned, please type them in the provided box(es)

|  | Not easy - 1 (18) | 2 (19) | 3 (20) | 4 (21) | Very easy - 5 (22) |
| --- | --- | --- | --- | --- | --- |
| 1. Food (1) |  |  |  |  |  |
| 2. Healthcare (2) |  |  |  |  |  |
| 3. Leisure/ exercise (3) |  |  |  |  |  |
| 4. ________ (type below) (4) |  |  |  |  |  |
| 5. _______ (type below) (5) |  |  |  |  |  |

Q45 Do you have internet at home?

- Yes (1)
- Yes, but it often gets disconnected (2)
- No (3)

Q46 Do you have access to one or more movie streaming services at home such as Netflix, Amazon Prime Video, or Disney Plus?

- Yes (1)
- No (2)

Q47 How often do you use these movie streaming services during an average day of the past week?

|  | 0 | 10 |
| --- | --- | --- |

| Hours () | 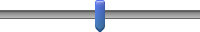 |
| --- | --- |

Q48 During an average day of the past week, how much time do you spend per average on social communication via smartphone, Facebook, and the many platforms?

|  | 0 | 10 |
| --- | --- | --- |

| Hours () | 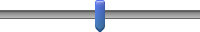 |
| --- | --- |

Q49 Thank you very much for your participation!   If you experience anxiety or emotional distress as a result of completing the survey, feel free to contact the CDC website for help under http://www.cdc.gov/coronavirus/2019-ncov/daily-life-coping/managing-stress-anxiety.html   We are also interested how our situations will be affected by the coronavirus crisis over time. Would it be ok to contact you again in a few months with some follow-up questions?   We greatly appreciate it!  
 
If so, please type your email address here:

We can also then send/ email you a copy of the research report (which might take a while).

________________________________________________________________

End of Block: Default Question Block
